# Effect of Rapid Urbanization in Mainland China on the Seasonal Influenza Epidemic: Spatiotemporal Analysis of Surveillance Data From 2010 to 2017

**DOI:** 10.2196/41435

**Published:** 2023-07-07

**Authors:** Hao Lei, Nan Zhang, Beidi Niu, Xiao Wang, Shenglan Xiao, Xiangjun Du, Tao Chen, Lei Yang, Dayan Wang, Benjamin Cowling, Yuguo Li, Yuelong Shu

**Affiliations:** 1 School of Public Health Zhejiang University Hangzhou China; 2 Key Laboratory of Green Built Environment and Energy Efficient Technology Beijing University of Technology Beijing China; 3 School of Public Health Shenzhen Campus, Sun Yat-sen University Shenzhen China; 4 Key Laboratory for Medical Virology Chinese Center for Disease Control and Prevention National Health Commission Beijing China; 5 School of Public Health, Li Ka Shing Faculty of Medicine The University of Hong Kong Pokfulam Hong Kong; 6 Department of Mechanical Engineering The University of Hong Kong Pokfulam Hong Kong

**Keywords:** seasonal influenza, attack rate, urbanization, urban population, human contact, agent-based model, influenza, seasonal flu, spatiotemporal, epidemic, disease transmission, disease spread, epidemiology, influenza transmission, epidemics

## Abstract

**Background:**

The world is undergoing an unprecedented wave of urbanization. However, the effect of rapid urbanization during the early or middle stages of urbanization on seasonal influenza transmission remains unknown. Since about 70% of the world population live in low-income countries, exploring the impact of urbanization on influenza transmission in urbanized countries is significant for global infection prediction and prevention.

**Objective:**

The aim of this study was to explore the effect of rapid urbanization on influenza transmission in China.

**Methods:**

We performed spatiotemporal analyses of province-level influenza surveillance data collected in Mainland China from April 1, 2010, to March 31, 2017. An agent-based model based on hourly human contact–related behaviors was built to simulate the influenza transmission dynamics and to explore the potential mechanism of the impact of urbanization on influenza transmission.

**Results:**

We observed persistent differences in the influenza epidemic attack rates among the provinces of Mainland China across the 7-year study period, and the attack rate in the winter waves exhibited a U-shaped relationship with the urbanization rates, with a turning point at 50%-60% urbanization across Mainland China. Rapid Chinese urbanization has led to increases in the urban population density and percentage of the workforce but decreases in household size and the percentage of student population. The net effect of increased influenza transmission in the community and workplaces but decreased transmission in households and schools yielded the observed U-shaped relationship.

**Conclusions:**

Our results highlight the complicated effects of urbanization on the seasonal influenza epidemic in China. As the current urbanization rate in China is approximately 59%, further urbanization with no relevant interventions suggests a worrisome increasing future trend in the influenza epidemic attack rate.

## Introduction

The influenza virus is mainly spread within close ranges rather than over long distances [1,2]. The distance from an influenza-infected individual may significantly affect the potential of influenza transmission. Social distancing is a well-recognized nonpharmaceutical intervention that could reduce the transmission of viruses from infected to susceptible individuals because of the increased physical distance between these individuals or reduced frequency of congregation in socially dense community settings such as schools or workplaces [3]. Urbanization is a complex phenomenon that is associated with population density in an urban area. In this study, urbanization rate, defined as the percentage of population living in an urban area, was used to characterize the urbanization process. Urbanization can worsen an epidemic through a variety of mechanisms [4]. Studies on low-income nations have clearly documented the association between epidemic severity and the combination of high-density living conditions, extreme poverty, and poor sanitation [5]. With respect to communicable diseases such as influenza, which is mainly spread within close ranges [1,2], the population concentrations in an urban area can lead to more rapid proliferation of infectious diseases [6]. Urbanization affects the seasonal epidemic transmission in highly urbanized countries [4,7]. In the United States with an urbanization rate of 83% in 2021 [8], cities in urban areas with higher population densities had a higher influenza transmission potential [7]. In Australia with an urbanization rate of 88% in 2021 [8], the peak influenza infection rate and peak prevalence increased steadily as the urban population density increased [4]. However, the impact of urbanization on the influenza epidemic in urban countries such as China with urbanization rates at 63% and India with urbanization rates at 35% in 2021, where about 70% of the population live, is less well-understood. In addition, in highly urbanized countries, urban living has been the norm in the last century. Therefore, the direct effects of further increases in the urban population density on the potential seasonal influenza epidemic might be less obvious [4]. In this study, attack rate, defined as the cumulative incidence rates of seasonal influenza during the study period, was used to characterize the seasonal influenza activities. Since urbanization will continue in urban countries in the near future, understanding the impact of urbanization on influenza transmission is essential for precise influenza prediction and prevention and for building a healthy society.

As a low-income country, China has experienced an unprecedented wave of rapid urbanization, as reported by the rapid increase in the urbanization rate from 17.9% in 1978 to 58.5% in 2017 [9]. The urbanization rate also varies significantly between provinces in China, thereby providing a unique opportunity to study the impact of urbanization on the influenza epidemic at different levels of urbanization within a single health care system. In China, urbanization has led not only to a rapid increase in the urban population density but also to a sharp decrease in the household size [10]. As households, schools (workplaces), and communities have been identified as the primary contexts of influenza transmission [11,12], the impact of urbanization on seasonal influenza in China may be complicated.

Dynamic models such as the susceptible-infected-recovered models have been developed to understand the dynamics of influenza transmission [7]. However, these models do not study the dynamics of influenza transmission at the individual level [13]. In highly urbanized countries, urban living has been the norm in the last century; thus, the susceptible-infected-recovered model could be used to study the impact of urbanization on influenza transmission [7]. However, the impact of urbanization on the influenza epidemic in urbanizing countries is complicated. In this study, to characterize the complicated impact of urbanization on human contact behaviors and population age structures in China, we used an agent-based model in which each individual or group of people was defined as an agent. The agent-based model based on human contact behavior has been used previously to model infectious disease transmission [11,14,15]. We also used similar models previously to successfully simulate influenza transmission in China [12,16].

In this study, we aimed to explore the effects of urbanization on seasonal influenza epidemic intensities in urban Mainland China (except Hong Kong, Macao, and Taiwan) according to the spatiotemporal analyses and modelling study of influenza surveillance data collected in Mainland China from 2010 to 2017.

## Methods

### Data Sources

We used weekly reports of influenza surveillance data from the Chinese National Influenza Center. The study data set was based on the number of specimens tested at 554 sentinel hospitals located in the urban areas of 31 provinces of Mainland China (including autonomous regions and municipalities). After excluding Tibet from the analysis due to the incompleteness of data, 30 provinces were evaluated. Although the surveillance data collection began in 2004, we focused on the period from April 1, 2010, to March 31, 2017, as the surveillance network was improved and expanded after the 2009 influenza A/H1N1 pandemic [17]. Shu et al [18] provide additional details about the Chinese influenza surveillance system. The data set of the influenza surveillance system in China included the number of visits at each hospital, number of cases of influenza-like illness (ILI), number of specimens tested, and number of laboratory-confirmed cases of influenza A (H1N1, H3N2, and pdmH1) and B (Yamagata and Victoria). ILI cases were identified based on a standard case definition: body temperature >38 °C, either cough or sore throat, and the absence of an alternative diagnosis. Several indicators were defined to characterize influenza activity in China. First, the ILI rate was defined as the number of ILI cases divided by the number of visits. Second, the influenza viral positive rate was defined as the number of laboratory-confirmed influenza cases divided by the number of specimens tested. In accordance with earlier studies [18-20], in this study, we defined a proxy for the weekly incidence rate (henceforth, incidence rate) of influenza, which is the multiplication of the ILI rate and the influenza viral positive rate. The incidence rate more precisely represents the incidence of influenza infection.

The influenza epidemic in Northern China intensifies in winter-spring months, while that in Southern China shows a semiannual cyclic pattern with clear peaks in both summer and winter [21,22]. We therefore defined an epidemiological annual cycle as the period from April 1 to March 31 of the following year [21], and we divided the annual cycle into summer months and winter-spring months, which included calendar weeks 14-39 (26 weeks in total) and weeks 40-13 of the following year (26 or 27 weeks in total), respectively [21]. The attack rate of the winter-spring or summer influenza epidemic in each province is defined as the cumulative weekly incidence rates during the half-year time (25 weeks in total): 12 weeks within the peak weeks since the median duration of an influenza epidemic wave is 15.6-22.5 weeks [21]. Province-level data on urbanization, urban areas, populations, household and school sizes, and number of students, schools, and factories from year 2010 to year 2016 were obtained from the China Statistical Yearbook [9], China City Statistical Yearbook [23], and National Bureau of Statistics of China [24].

### Modelling

We used an agent-based model to simulate the influenza transmission dynamics in provinces with different urbanization rates. Our simulation strategy is similar to that reported previously in the literature [11]. Similar models have been used to simulate influenza transmission in Beijing [25] and Hong Kong [16]. In this study, we simplified the model and considered 4 types of indoor spaces: homes, schools, workplaces, and communities (ie, public spaces). These 4 types of indoor spaces are known as the primary contexts of influenza transmission [11,12]. Populations were divided into 3 groups: workers, students, and others.

**Figure 1:**
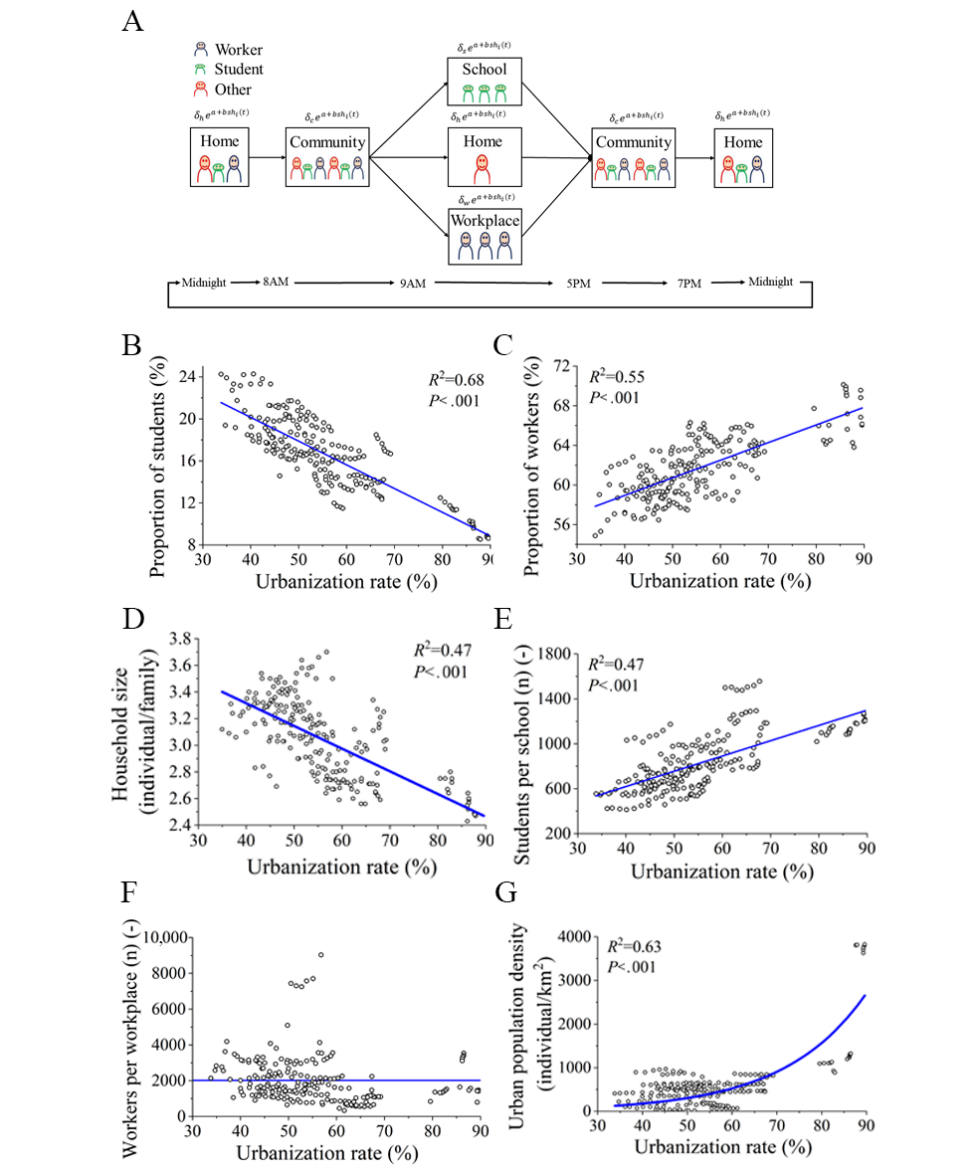
Individual routines and population distribution data in Mainland China between 2010 and 2016. (A) Flowchart of individual hourly routines. (B) Changes in the proportion of students with increasing urbanization. (C) Changes in the proportion of the workforce with increasing urbanization. (D) Changes in the household size with increasing urbanization. (E) Changes in the school size distribution with increasing urbanization. (F) Changes in the workplace size distribution with increasing urbanization. (G) Changes in the urban population density with increasing urbanization rates.

Figure 1A presents the assumed typical daily movements of 3 groups of people. Although real human movement patterns are much more complicated, we considered the simulated movement pattern to represent a general daily schedule. The time step in the simulation was set to 1 hour. For each case, the simulation was performed with 100 replications to account for randomness in the status of each individual (infectious, susceptible/removed). Differences in human movement behaviors between weekdays and weekends were not considered. The percentage of students decreased significantly with urbanization (Figure 1B). The number of workers (*N_W_*) was estimated based on the number of people aged between 18 and 60 years (*N_18-60_*), the number of students in universities (*N_SU_*), and the unemployment rate (*R_u_*) as *N_W_* = (*N_18-60_* – *N_SU_*)∗(1 – *R_u_*). The percentage of workers increased significantly with urbanization (Figure 1C). The remaining populations were classified as others. Influenza transmission is affected by the number of students, workers, and others, as well as by the distributions of these populations in each indoor space. The province-level distribution of household sizes at different urbanization levels is shown in Figure 1D. Notably, the school size increased with urbanization (Figure 1E). As we did not observe a significant linear relationship between urbanization and the number of workers per workplace (Figure 1F), we adopted a mean value of 2020 workers per workplace at all urbanization levels. With urbanization, the urban population density increased exponentially (Figure 1G), and it is reasonable to believe that population contact in the community would also increase.

Here, we defined *Ii* as 1 if individual *i* were infectious and 0 otherwise. The force of infection per hour experienced by individual *i* by 1 infected person in a home, community, school, and workplace was then calculated as *I_k_*δ_h_e*^a+bshi(t)^*, *I_k_*δ_c_e*^a+bshi(t)^*, *I_k_*δ_s_e*^a+bshi(t)^*, and *I_k_*δ_w_e*^a+bshi(t)^*, respectively, where n_hi_ is the household size of individual *I*; n_ci_ is the mean number of people in the community that individual *i* would contact per hour, which was assumed to be in proportion to the urban population density [25]; n_si_ (n_wi_) is the mean number of individuals that individual *i* would contact in school (workplace) per hour if he/she were a student (worker), which was assumed to be constant in different provinces and n_si_=9, n_wi_=4 [26]; and *a* and *b* are parameters used to adjust the impact of specific humidity, *sh*(t), at time t on influenza transmission. For this study, *a*=0.788 and *b*=–180 [27], while δ_h_, δ_c_, δ_s_, and δ_w_ indicate the influenza transmission coefficients in a home, community, school, and workplace, respectively. We assumed that δ_h_:δ_c_:δ_s_:δ_w_ = 6:1:12:6 [11], as children are known to have high clinical attack rates of influenza [28,29]. The absolute influenza transmission coefficients in homes, communities, schools, and workplaces (δ_h_, δ_c_, δ_s_, and δ_w_, respectively) are always unknown, and these are mainly determined by the antigenic evolution of the influenza virus and thus vary across years [7]. Therefore, we relied on our model approach to back-calculate the transmission coefficients. For each year, we selected the surveillance data in 1 province and used the least squares method to determine the influenza transmission coefficient parameter value in that year. Then, we used the back-calculated transmission coefficients to simulate the influenza transmission in other provinces and compared them with surveillance data to test our model. The simulation duration of influenza transmission was set to 8 months from November 1, 2013, to June 30, 2014. The time step was set to 1 hour. Each simulation under different settings was conducted 100 times to improve the reliability of the results. The latent and infectious periods were set to 2 and 4 days [30], respectively.

There were 2 types of settings in our simulation. When we simulated infection spread in different provinces, the real parameters from each province were used. When we simulated infection spread at different urbanization levels, the ideal parameters obtained by data fitting based on real parameters from all provinces were used. In this setting, Beijing climate data were used to eliminate the impact of climate differences. The detailed model structure and parameter estimation are shown in Multimedia Appendix 1 [11,12,27,30-34].

### Spatial Analysis of the Influenza Surveillance Data by Province

The spread of influenza is influenced by climate [35]. As China is a climatologically diverse country, we cannot directly compare the province-level intensities of the influenza epidemic. To minimize the influence of climate on the analysis, we attempted to compare the epidemic attack rates in provinces with similar climates by using the following 2 methods. First, we analyzed the attack rates of the influenza epidemic in 15 provinces in Northern China that are situated in the temperate climate zone. Second, there is no significant latitude gradient with respect to province-level urbanization in China. Therefore, we classified the provinces according to the urbanization rate. The province urbanization rates ranged from 33.8% to 89.6%, and therefore, we classified the provinces according to the following urbanization rate ranges: <40%, 40%-50%, 50%-60%, 60%-70%, and >70%. The urbanization within each range was defined as the mean urbanization level of the provinces within this range. The influenza epidemic attack rate was also defined as the mean attack rate in the provinces within each urbanization range. Using year 2013 as an example, the provinces within different urbanization ranges are shown in Figure S2 of Multimedia Appendix 1. The mean latitudes of the provinces with urbanization levels <50%, 50%-60%, 60%-70%, and >70% were 31.8, 35.7, 30.4, and 36.8 °N, respectively; the variance in the latitude was narrow within each urbanization range. Therefore, the influence of climate could be minimized. Last, binomial fitting was used to quantify the observed U-shaped relationships between the province-level influenza epidemic attack rates and urbanization rates by the following equation: y = ax^2^+bx+c, where y represents the influenza epidemic attack and x represents the urbanization rate. The least squares method was used to estimate the unknown parameters a, b, and c. The turning point of the urbanization rate was –b/2a.

### Ethical Considerations

Data in this study are from public database, ie, Chinese National Influenza Centre [36]. We did not make any interventions in the study.

## Results

### Spatiotemporal Analysis of the Influenza Surveillance Data

**Figure 2:**
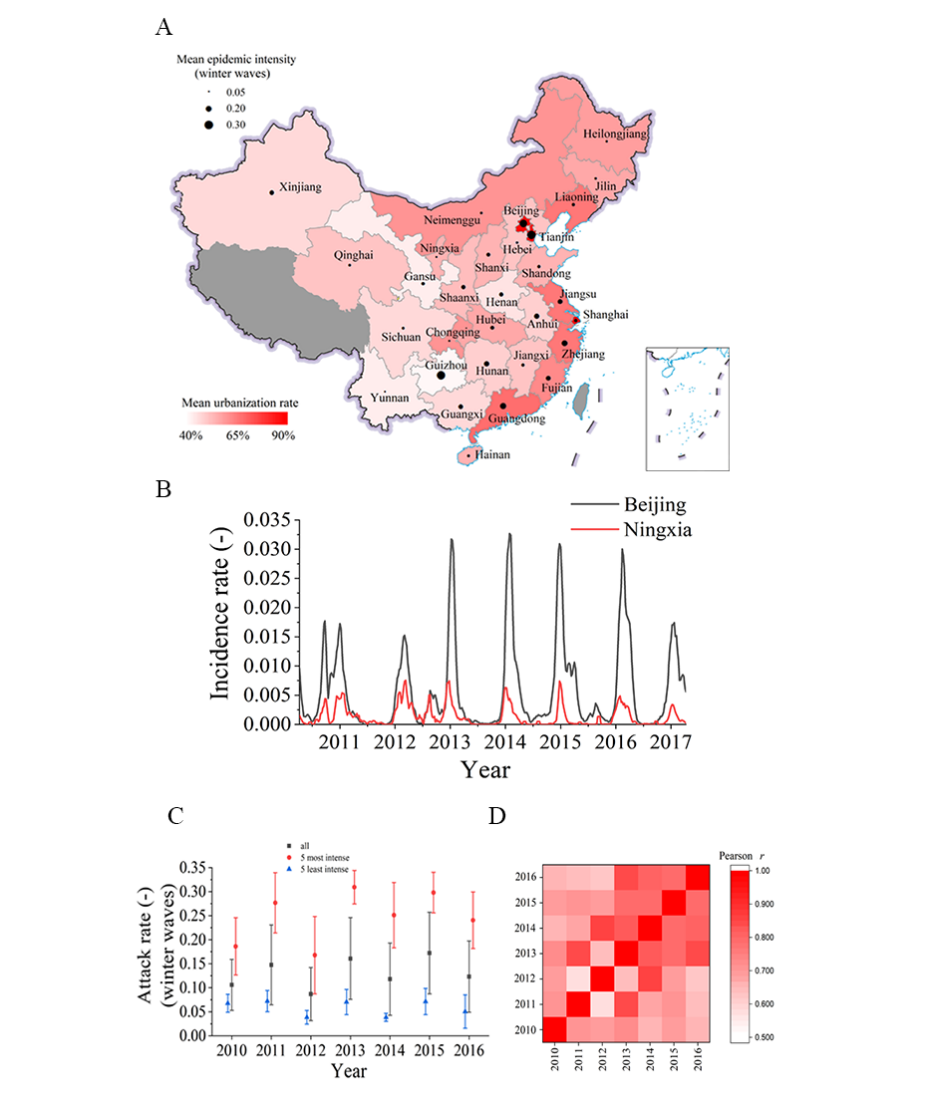
Systematic differences in the seasonal influenza epidemic attack rates in the winter-spring waves across provinces in Mainland China according to surveillance data between 2010 and 2016. (A) Mean influenza epidemic attack rates (black circles) and urbanization rates (colored bars) in 30 Chinese provinces; grey areas indicate a lack of data. (B) Time-series influenza incidence rates in Beijing and Ningxia provinces. (C) Influenza epidemic attack rates in 5 provinces with the most or least intense epidemic and in all provinces across epidemiological years, where error lines represent 95% CIs. (D) Pearson correlation coefficients of the epidemic attack rates in 30 provinces between epidemiological years.

During the study period between 2010 and 2016, differences in the mean epidemic attack rate followed a geographic pattern, with the highest intensive epidemic detected in both the most and least urban regions of Beijing (mean urbanization, 86%) and Guizhou (mean urbanization, 39%), respectively. The lowest epidemic attack rate was observed in moderate urban regions such as Ningxia (mean urbanization, 53%) and Chongqing (mean urbanization, 58%; Figure 2A). In addition, the differences persisted over time, that is, some provinces had consistently more intense epidemic than others year after year. The influenza incidence rates in Beijing were always higher than those in Ningxia (Figure 2B). For these 5 provinces with the most or least intense epidemic, the mean epidemic attack rates followed a similar trend over time as the mean epidemic attack rates in all 30 provinces (Figure 2C). The Pearson correlation coefficients of the epidemic attack rates in the 30 provinces between any 2 epidemiological years were higher than 0.5 (Figure 2D), indicating that the differences in the epidemic attack rates among provinces persisted over time. There was no significant linear relationship between the urbanization rate and influenza surveillance intensity (*P*=.64) (Figure S3 of Multimedia Appendix 1).

### Modelling Study

**Figure 3:**
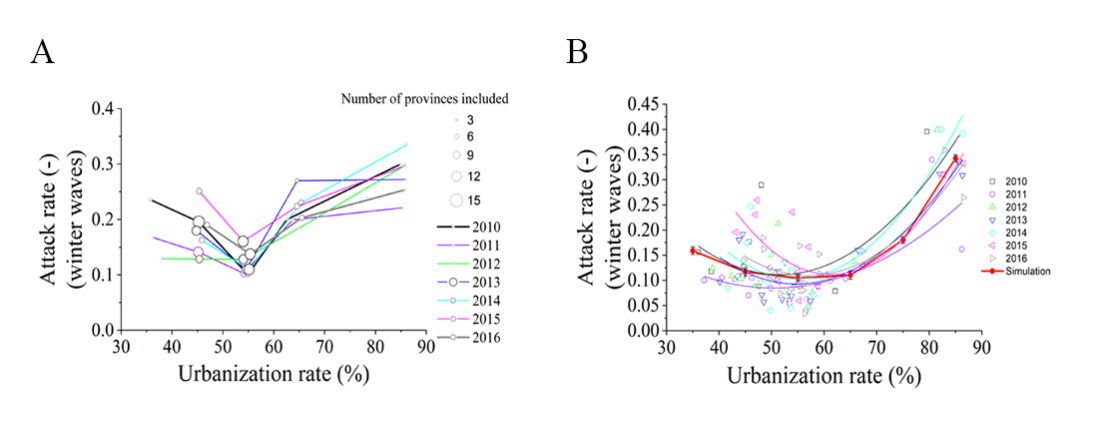
Determination of a U-shaped relationship between the influenza epidemic attack rates and urbanization rate in China. Data are presented by (A) clustering of 30 provinces by the urbanization rate and (B) province-level data in 15 provinces in Northern China. The curves are the binomial fitting of the data in 2010-2016. The red line indicates the agent-based model simulation results, and the error bars indicate the 95% CIs of the 100 replications.

We hypothesize that these patterns of epidemic attack rates are due to urbanization. The categorization of the provinces by urbanization rates revealed that the correlation between the influenza epidemic attack rate and urbanization rate yielded a U-shaped curve throughout the study period, with a turning point at about 55% urbanization (Figure 3A). From our binomial-fitter curves, we also found U-shaped relationships between province-level influenza epidemic attack rates and urbanization rates in 15 provinces in Northern China, and we consistently observed the lowest attack rates in provinces with urbanization rates of 50%-60% (Figure 3B and Table 1). The agent-based model that we built in this study also revealed U-shaped relationships between province-level influenza epidemic attack rates and urbanization rates (red line in Figure 3B). The sensitivity analyses of the key parameters in the agent-based model are shown in Figure S4 of Multimedia Appendix 1.

**Table 1:** Binomial fitting of the province-level epidemic attack rates in the 15 provinces in Northern China.

Epidemiological year	*R* ^2^	*P* value	Urbanization rate turning point (%)
2010	0.61	.004	52
2011	0.51	.01	50
2012	0.76	<.001	50
2013	0.77	<.001	55
2014	0.79	<.001	55
2015	0.67	.001	61
2016	0.68	.001	57

**Figure 4:**
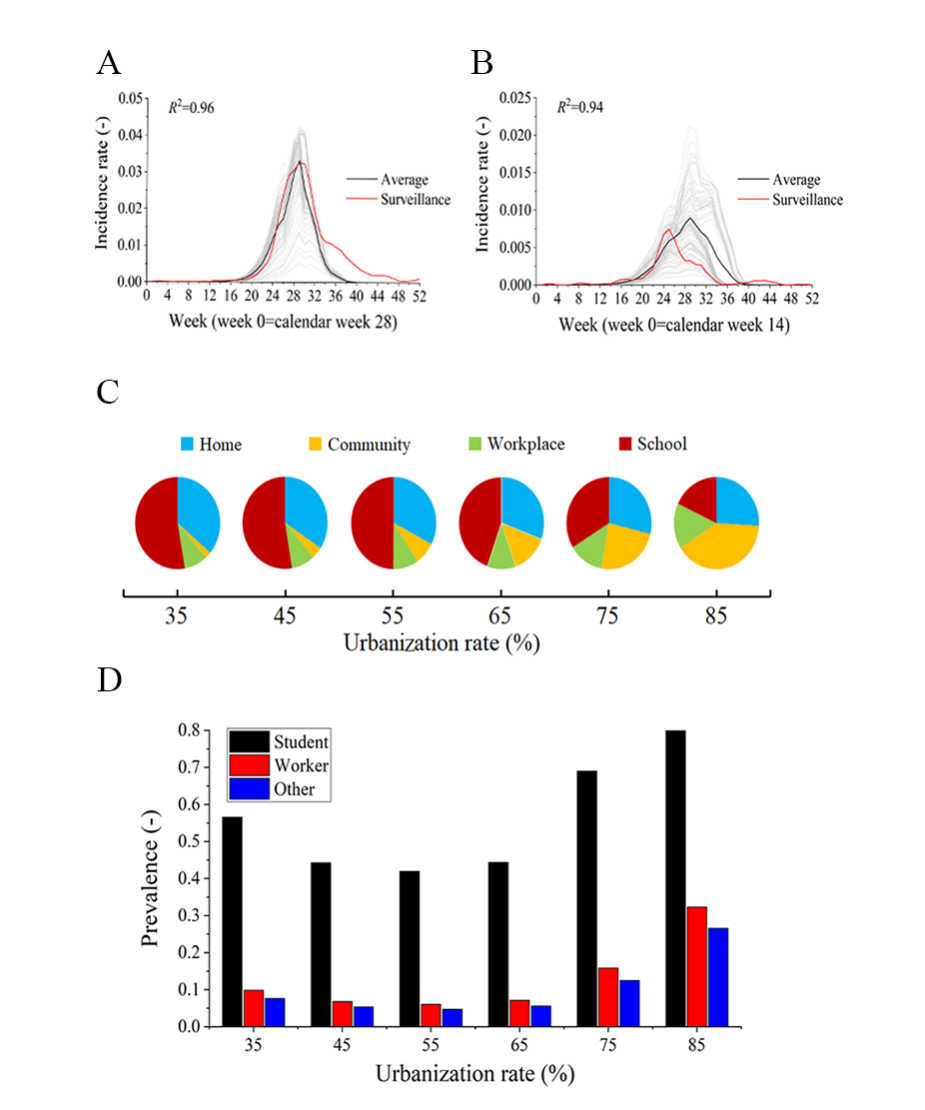
Simulated influenza epidemic in provinces with different urbanization rates. Simulated incidence rates in (A) Beijing and (B) Jilin. (C) Simulated contributions of each setting to influenza transmission according to the urbanization rate. (D) Influenza prevalence among students, workers, and others according to the urbanization rate.

Next, we simulated the influenza transmission to quantify the U-shaped relationship observed in Figure 3. As demonstrated using 2013 as an example, we selected surveillance data from Beijing for back-calculation of the unknown transmission coefficients (Figure 4A, *R*^2^=0.96). The back-calculated transmission coefficients and our model simulated the influenza transmission dynamics in Jilin well (Figure 4B, *R*^2^=0.94), demonstrating that the models could be used to simulate influenza transmission in China. Next, we used the model to simulate influenza transmission in provinces with different urbanization rates. The model also yielded a U-shaped association between the urbanization rate and influenza epidemic attack rate (Figure 3B), with a turning point at 55% urbanization.

Taking the simulation in Beijing and Jilin as an example, the agent-based model built in this study well-fitted the influenza surveillance data (Figure 4A and 4B). Thus, using the agent-based model built in this study, we simulated the contributions of different locations and people in influenza transmission with different urbanization rates. Figure 4C presents the percentage contributions of the 4 studied locations with different urbanization rates to influenza transmission. As the urbanization rate increased from 35% to 85%, the contributions of each location to influenza transmission increased from 2.7% to 41.8%: the contribution of workplaces increased from 8.3% to 13.4%, but that of homes decreased from 38.9% to 27.4% and that of schools decreased from 50.2% to 17.4%. Given the relatively frequent number of contacts and the high infection risk per contact in schools, our simulation results demonstrate that students face a much higher infection risk than workers (relative risk 5.4, SD 1.7) and others (relative risk 6.8, SD 2.2; Figure 4D).

## Discussion

To the best our knowledge, this study is the first to explore the effect of urbanization on the attack rate of the seasonal influenza epidemic in a country experiencing an unprecedented rapid rate of urbanization. This study was made possible by the availability of census data and influenza surveillance data. Our analyses revealed a U-shaped relationship between the urbanization rate and influenza attack rate, with a turning point at around 55% urbanization rate. We used an agent-based mathematical model based on hourly human contact behaviors to explore the 2 main primary mechanisms responsible for the observed trend. We found that an increase in the urban population density (Figure 1G) increased the number of close contacts between people in communities and thus enhanced the transmission of influenza in public areas. Further, decreases in household sizes (Figure 1D) and percentages of students (Figure 1B) reduced the rates of influenza transmission in homes and schools, respectively. The net effects of these 2 mechanisms provide a simple but reasonable explanation for the observed U-shaped trend in the influenza epidemic attack rate over the 7-year study period. Further confirmation of this U-shaped trend would have significant implications for the influenza epidemic in China. In addition, although this study analyzes the influenza data in China, the methods may also be applicable in other urbanizing countries and for other respiratory infections such as COVID-19, measles, and Middle East respiratory syndrome because indoor environments such as households, schools (workplaces), and communities have always been identified as the primary context of infection transmission [37,38].

The epidemic attack rate is an important parameter for the development of medical surge capacities and public health systems, including primary care facilities. This is particularly important for influenza, as a vaccine specific for a new pandemic virus might not be widely available for up to 6 months based on the current vaccine production technology [3]. Previous studies have proposed that socioeconomic factors may affect both the influenza epidemic and pandemic [4,7,39]. Our study also demonstrates regional differences in the intensity of the influenza epidemic across China, which suggest that seasonal influenza interventions should vary among provinces. We found that the most intense influenza epidemic occurred in the most and least urbanized provinces, suggesting that increased attention should be directed to influenza control in the latter provinces, which are also economically underdeveloped. Previous analyses of the history of infectious diseases and human populations have suggested that the host population density is a critical determinant of the establishment and persistent endemicity of a pathogen in a population. Accordingly, urban aggregations play an important role in maintaining infectious disease endemics [40]. As people have become increasingly concentrated in urban areas, and particularly in large cities, influenza viruses could potentially be spread more easily nowadays. Moreover, large cities always serve as hubs in intercity travel networks, which are linked by high-speed rail in China. These hubs could also influence the spread of the influenza epidemic among cities. These potential trends associated with urbanization pose a challenge to future influenza interventions.

There are at least 2 policy implications to build healthy cities in the aspect of infection control. The first is that influenza vaccine recommendations in China should speed up since vaccine coverage is only approximately 2% in China [41], especially in provinces with urbanization rates greater than 60%. In this study, we found a U-shaped relationship between the influenza epidemic attack rate and urbanization rate, with a turning point between 50% and 60% urbanization. In China, the recent urbanization rate is approximately 60%, and a large number of provinces are currently at the turning point in the U-shaped trend curve. As further urbanization is expected, the trajectory of the influenza epidemic intensity in those provinces in the near future would be worrisome if no interventions were made because of the nonlinear increase in the strain placed on the medical infrastructure [4]. The second is that nonpharmaceutical interventions implemented to control the influenza epidemic and even pandemic should vary by province since this study demonstrates regional differences in the intensity of the influenza epidemic across China. This study shows that in low urbanized regions, influenza transmission mainly occurs in the schools, while in highly urbanized regions, influenza transmission mainly occurs in the community. These findings are well supported by the observed decreasing effectiveness of school closure with increasing urbanization in China [42]. Thus, in highly urbanized regions, ventilation and surface cleaning/disinfection in public places may be effective, while in low urbanized regions, school closures should be recommended.

Parameters in the simulation determined the infectious disease transmission (eg, attack rate). Some parameters such as the time schedule of workers and students and number of students per class were obtained from the literature or real statistical data. However, other parameters such as the daily numbers of the contacted students in each class and the number of workers in each office were estimated and assumed. We adjusted these parameters to minimize the difference between the simulation results and the real infection spread. Moreover, we performed a sensitivity analysis for 10 parameters to show how these parameters influence the infection spread (Figure S4 of Multimedia Appendix 1).

This study suggests a number of limitations and possibilities for future research. First, our model did not consider differences in the medical service levels. Highly urbanized areas have access to better medical services, which would enable a more rapid cure of infectious cases and better control of the influenza epidemic. However, approximately 90% of the seasonal influenza cases remain unreported [43], and infectious cases are mostly transmissible during the incubation period and during 2-3 days with clinical symptoms. Therefore, the medical service level would have a very limited impact on the influenza epidemic. Second, our model did not consider differences in the influenza vaccine coverage because this rate has increased only gradually to approximately 2% of the Chinese population over the past 15 years [41]. This low influenza vaccination rate would have a very limited influence on an epidemic. Third, the results of this study cannot be generalized to rural areas since there is a lack of rural-related data. Fourth, this study assumes that all types of agents (eg, worker, student) have a constant time schedule and all offices and classrooms in a province have the same setting. Therefore, the random scenarios, which could be referred to as a dynamic network, were ignored. Finally, higher resolution analyses of the influenza epidemic, such as those conducted at a city level, would provide more accurate information, as cities are the principal locations of influenza transmission between humans [7]. However, our relatively coarse data set did not allow us to evaluate the influenza patterns at the city or hospital level, even though the surveillance data from 4 of the 30 studied provinces were based on single cities (Beijing, Tianjin, Chongqing, and Shanghai).

In conclusion, we found a U-shaped relationship between the influenza attack rates and urbanization rate in provinces of the urbanizing mainland China, with a turning point between 50% and 60% urbanization. This finding is different from that reported in other studies in urbanized countries. Our study poses the potential challenges of the expected further urbanization process in China on population health and health infrastructure. Further, our results will be critical for precise influenza prediction and prevention.

## Appendix

Multimedia Appendix 1 Supplementary data.

## Abbreviations

ILI: influenza-like illness

## Data Availability

Descriptions of the model structure and the parameters included in the model are available in Multimedia Appendix 1. Detailed influenza surveillance data are available from the corresponding author on reasonable request. The model code is available from the author’s GitHub site [44].
