# Supplementary material for: Effect of Rapid Urbanization in Mainland China on the Seasonal Influenza Epidemic: Spatiotemporal Analysis of Surveillance Data From 2010 to 2017
Source: JMIR Public Health Surveill. 2023 Jul 7;9:e41435. doi: 10.2196/41435 (PMC10362421; doi:10.2196/41435)
Supplement: Multimedia Appendix 1 [file publichealth_v9i1e41435_app1.docx]

**Multimedia Appendix 1**

**1. Detailed model structure, parameter selection**

**Modelling**

We used C++ by Qt creator 5.14, an open-source software ([www.qt.io](http://www.qt.io)) for programming. In the simulation, firstly, all related data (e.g. climate data, classroom and office assignment) were loaded and set. Then two steps including people movement (which person stayed at which indoor environment at what time, with time resolution of one hour) and infection spread in each indoor environment were conducted.

The simulation duration of influenza transmission was set to 8 months from 2013 November 1 to 2014 June 30. The time step was set to 1 h. Each simulation under different settings was conducted 100 times in order to improve the reliability of the results. Latent and infectious periods were set to 48 and 96 h, respectively[28, 38-39]. Because we only focused on one influenza season, therefore, we hypothesized that recovered people cannot be infected again.

There were two types of settings in our simulation. When we simulated infection spread in different provinces (Figures 4A and 4B), the real parameters from each province were used. When we simulate infection spread under different urbanisation levels (Figures 4C, and 4D), the ideal parameters obtained by data fitting based on real parameters from all provinces were used. In this setting, climate data of Beijing was used in order to eliminate the impact brought by climate difference.

Settings for population, location, and climate are listed below. Province-level data on urbanisation, urban areas and populations, household and school sizes and the numbers of students, schools and factories from year 2010 to 2016 were obtained from the *China Statistical Yearbook,* available at: <http://www.stats.gov.cn/tjsj/ndsj/> and *China City Statistical Yearbook*, available at: [http://www.mohurd.gov.cn/xytj/ tjzljsxytjgb/](http://www.mohurd.gov.cn/xytj/%20tjzljsxytjgb/). Meteorological data were obtained from China Meteorological Administration (CMA).

*Population*

The total population in each simulation was set to 1,000,000. People were divided into three categories: student, worker, and others. Prevalence data of influenza was from the private and public hospitals located in urban areas, which means the influenza data in rural area was unknown. Therefore, the number of students, workers, and total population in urban area have to be obtained.

(1) Student

Real data from census showed the total number of students in primary schools, middle schools (includes secondary vocational schools), high schools (includes higher vocational schools), special education schools, and universities. We hypothesized that both urban and rural areas have the same percentage of students in primary, middle, high, and special education schools. However, all students in universities belong to urban areas because almost all universities are located in cities. Therefore, the percentage of students in urban areas (*P_S_*) is calculated by: *P_S_={[(N_u_/N)(N_sp_+N_sm_+N_sh_+N_se_)+N_su_]/N_u_}**100%, where $N$ and $N_{u}$ is the total population in the province and its urban area, N_sp_, N_sm_, N_sh_, N_se_ N_su_ and N_su_ are the total number of students in the primary schools, middle schools, high schools, special education schools, and universities in the province (both urban and rural areas), respectively. As shown in Figure 1B, the relationship between percentage of students in the urban area and urbanisation rate (*R_u_*) obeys the equation of *P_s_=0.29195-0.226R_u_*.

(2) Worker

People in urban areas aged from 18 to 60 excluding those who are students in the universities and unemployed were regarded as workers. We hypothesized that the percentage of people aged from 18 to 60 (*P_18-60_*) in both urban and rural areas are the same. Therefore, the percentage of workers in the urban area (*P_w_*) is calculated by: *P_w_={[(P_18-60_*N_u_-N_su_)*(1-P_ue_)]/N_u_}**100%, where *P_ue_* is the unemployment rate of the province. As shown in Figure 1C, the relationship between percentage of workers in the urban area and urbanisation rate obeys the equation of *P_w_*=0.51831+0.178*R_u_*.

(3) Other

Those people who are neither students nor workers are regarded as the others. The percentage of the others (*P_o_*) is calculated as *P_o_=*(*1-P_w_-P_s_*)*100%.

*Location*

All people are located at four places based on their commutes (Figure 1A): home, workplace, school, and community.

1. Home

All people stay at homes during 19 pm to 8 am of the next day. Those who are not students and workers stay at home during 9 am to 17 pm. The household size (*N_h_*) changes with urbanisation rate (*R_u_*) according with the equation of *N_h_*=3.997-1.700*R_u_*, where *R_u_* is from 0% to 100%. People are randomly assigned to homes. The influenza transmission coefficient in a home *β_h_*=1.63*10^-3^.

1. School

All students stay at schools from 9 am to 17 pm. The school size changes with the urbanisation rate according with the equation of *N_s_*=[1360.5**R_u_*+75.658], where *N_s_* is the number of students of each school. Each class has no more than 50 students, therefore, each school has the number of class of [*N_s_*/50]. Each student on average contacts 9 students per day. Considering most students prefer contacting the students in the same class, we hypothesized that only 1% of contacted students come from other classes. We also hypothesized that the friends of each students are unchanged, which means each student has the same friend connections during per simulation. All students are randomly assigned to schools. The influenza transmission coefficient in a school *β_s_*=3.26*10^-3^.

1. Workplace

All workers stay at workplaces from 9 am to 17 pm. The company size is hypothesized to be a constant (100 workers per company), which means it is not changed with urbanisation rate. Each office has no more than 15 workers, therefore, there are 7 offices per company. Considering most workers prefer contacting colleagues in the same office, we hypothesized that only 2% of contacted workers come from other offices. We also hypothesized that the colleagues of each worker are unchanged, which means the colleague connections are fixed during per simulation. Workers are randomly assigned to workplaces. The influenza transmission coefficient in a workplace *β_w_*=1.63*10^-3^.

1. Community

All people stay at communities from 8 am to 9 am and from 17 pm to 19 pm. The community size changes with urbanisation rates. In the simulation, we hypothesized that the average community size in Beijing is 90. And community size ($N_{c}$) in other provinces accords with the equation of *N_c_*=[0.5+90*(1.056^100*Ru^/1.056^100*86%^)], where 86% is the urbanisation rate of Beijing. All people in community are randomly distributed, which means each person can be located at any community during the time from 8 am to 9 am and from 17 pm to 19 pm. The influenza transmission coefficient in a community *β_c_*=2.72*10^-4^.

*Climate change*

Virus viability is strongly influenced by climate (e.g. temperature and humidity). In this study, we referred the results of Pei et al[25]. to set the impacts on the force of infection by climate. The study showed that specific humidity influences the force of infection with the equation of *e^a+b*SH^*, where a and b are suggested to set to be 0.788 and -180, respectively [25]. Specific humidity (*SH*) is calculated by Equation S1 [40].

*SH*={0.6112*exp*[17.67T/(T+243.5)]*[21.674*RH/(T+273.15)]}/1000*ρ_a_(T)* (S1)

where *T* is temperature (K), RH% is relative humidity, *ρ_a_(T)* with the unit of g/m^3^ is density of the air under temperature T.

Based on daily data on temperature and relative humidity, the coefficient on the force of infection by climate during each day in the 15 provinces in North China are calculated (Table S1).

*Modelling step size*

Because the human daily routine was set by hour in the study (Figure 1A), the time step in the simulation was set to 1 hour. For each case, the simulation was performed with 100 replications to account for randomness in the status of each individual (infectious, susceptible or removed).

Table S2 summarized the parameters in the model.

**Table S1.** The averaged coefficient on the force of infection by climate

| Province | Averaged coefficient on the force of infection by climate | | | | | | | |
| --- | --- | --- | --- | --- | --- | --- | --- | --- |
|  | Nov. | Dec. | Jan. | Feb. | Mar. | Apr. | May | Jun. |
| Beijing | 1.62 | 1.85 | 1.86 | 1.71 | 1.61 | 1.21 | 1.04 | 0.50 |
| Gansu | 1.69 | 1.86 | 1.96 | 1.81 | 1.73 | 1.36 | 1.36 | 0.86 |
| Hebei | 1.56 | 1.79 | 1.79 | 1.68 | 1.50 | 1.13 | 0.95 | 0.47 |
| Henan | 1.18 | 1.56 | 1.56 | 1.33 | 1.12 | 0.73 | 0.66 | 0.35 |
| Heilongjiang | 1.58 | 1.88 | 2.03 | 1.97 | 1.70 | 1.50 | 0.93 | 0.43 |
| Jilin | 1.57 | 1.87 | 1.91 | 1.91 | 1.66 | 1.50 | 0.94 | 0.49 |
| Liaoning | 1.47 | 1.78 | 1.84 | 1.81 | 1.55 | 1.30 | 0.88 | 0.41 |
| Inner Mongolia | 1.75 | 1.93 | 2.00 | 1.96 | 1.84 | 1.61 | 1.27 | 0.75 |
| Ningxia | 1.66 | 1.86 | 1.96 | 1.72 | 1.70 | 1.25 | 1.36 | 0.80 |
| Qinghai | 1.90 | 2.02 | 2.05 | 1.97 | 1.89 | 1.69 | 1.58 | 1.13 |
| Shandong | 1.27 | 1.58 | 1.54 | 1.49 | 1.26 | 0.90 | 0.76 | 0.35 |
| Shanxi | 1.59 | 1.86 | 1.91 | 1.65 | 1.62 | 1.14 | 1.13 | 0.66 |
| Shaanxi | 1.24 | 1.67 | 1.76 | 1.46 | 1.35 | 0.88 | 0.86 | 0.51 |
| Tianjin | 1.48 | 1.70 | 1.67 | 1.59 | 1.36 | 1.05 | 0.86 | 0.39 |
| Xinjiang | 1.60 | 1.74 | 1.87 | 1.88 | 1.63 | 1.51 | 1.37 | 0.95 |

**Table S2**. Summary of parameters in the model.

| Parameter | Description | Value | Source |
| --- | --- | --- | --- |
| **N_I_** | Number of the initial infected people | 20 | Assumed |
| **δ_h_** | Influenza transmission coefficient in a home | 0.0013 | Estimated and Ferguson et al., 2005 [11] |
| **δ_c_** | Influenza transmission coefficient in a community | 0.00021 |  |
| **δ_w_** | Influenza transmission coefficient in a workplace | 0.0013 |  |
| **δ_s_** | Influenza transmission coefficient in a school | 0.0026 |  |
| ***a*** | A coefficient brought by climate | 0.788 | Pei et al., 2018 [25] |
| ***b*** | A coefficient brought by climate | -180 |  |
| **N_OC_** | Number of contacted workers in each office per day | 4/day | Estimated |
| **N_O_** | Number of workers in each office | 15 | Estimated |
| **N_C_** | Number of workers in each company | 100 | Estimated |
| **P_O_** | Probability of workers contacting workers in other offices | 2% | Assumed |
| **N_CM_** | Number of people in each community in Beijing (urbanisation rate = 86%) | 90 | Estimated |
| **N_CLC_** | Number of contacted students in each class per day | 9/day | Estimated |
| **N_CL_** | Number of students in each class | 50 | Zhang et al., 2020[41] |
| **N_Smin_** | Minimum number of students per school | 350 | Statistics |
| **N_Smax_** | Minimum number of students per school | 1600 |  |
| **P_C_** | Probability of students contacting students in other offices | 1% | Assumed |
| **T_Wstart_** | Time for work/class | 9 am | Zhang et al., 2018 [12] |
| **T_Wend_** | Time for being off work/school | 5 pm |  |
| **T_HL_** | Time for leaving home in the morning | 8 am |  |
| **T_HG_** | Time for going home at night | 7 pm |  |
| **T_L_** | Latent period | 48 h | Mills et al., 2004 [28] |
| **T_I_** | Infectious period | 96 h |  |

During the initial parameter setting, all real climate data (e.g. temperature, relative humidity, specific humidity) from each day during the simulation period was loaded. All personal parameters including home ID (which home lives in), community ID (which community prefers to), occupation (worker, student, others), office and company ID (which office and company works in), class and school ID (which class and school learn in), and initial infection status (susceptible, infected, and removal) were set. All personal parameters mentioned above were randomly set based on real percentage distribution. After all parameters setting, the simulation would move to the infection and move steps. During the infection step, all people are assigned to each environment (home, office/company, class/school, and community). Susceptible people have probability to be infected if they contact with the infected. During the move step, all people move from one environment to another environment based on the specific pattern (Figure 1A). For example, students will leave home to school/class at 8 am. The time interval of the simulation is set to one hour, therefore, one day includes 24 circulations of infection and move steps.

**Figure S1.** Programming structure of the simulation

**2. Classification of provinces by urbanisation level.**





**Figure S2**. Classification of the provinces by urbanisation rates.

**3.** **Urbanisation rates and the weekly average number of specimens tested**

During the study period ranging from 1 April, 2010 to 31 March 2017, an average of 246 specimens were tested weekly per province. The highest average sampling rate was recorded in Guangdong province, at 530 per week, while the lowest rate was observed in Hainan province, at 104 per week. This level of sampling corresponds to the testing of an average of 3.0 respiratory samples per 10,000 residents per year in China.


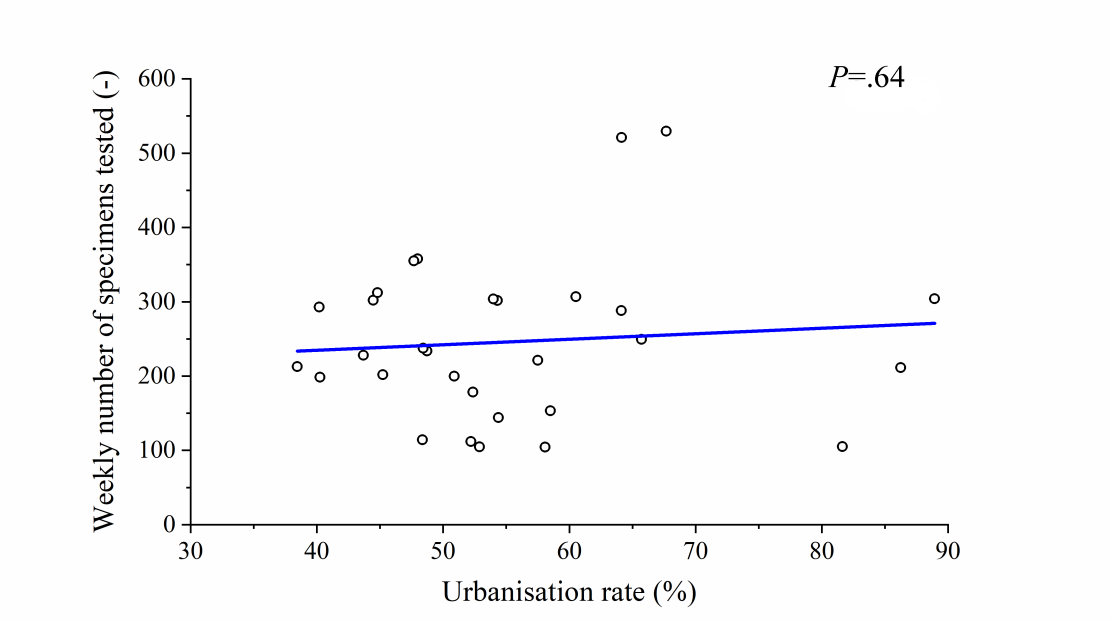


**Figure S3.** Intensity of influenza surveillance sample collection in provinces classified by urbanisation rate.

**4.** **Sensitivity analysis**





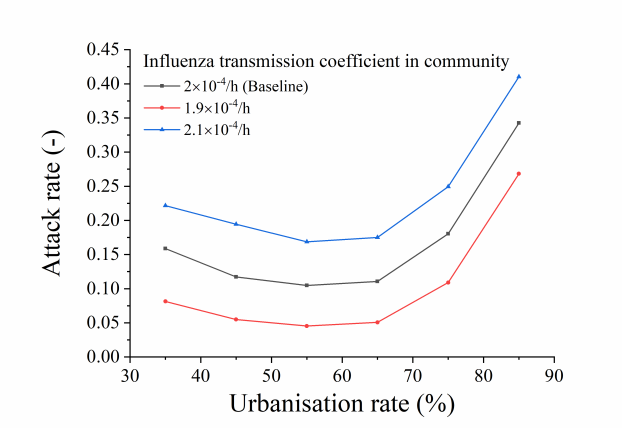


(A) (B)







(C) (D)


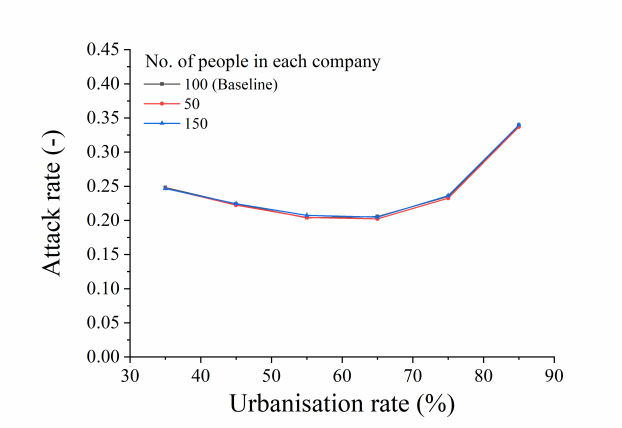

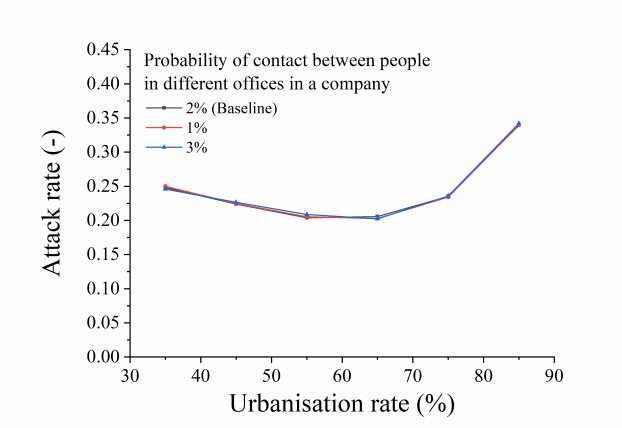


(E) (F)







(G) (H)





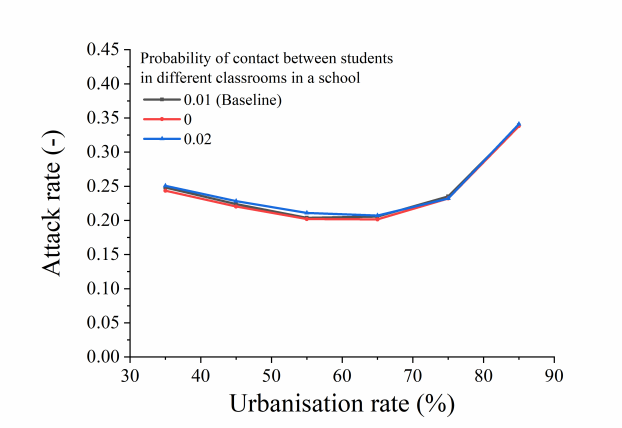


(I) (J)

**Figure S4.** Sensitivity analysis of the key parameters in the model. (A) influenza transmission coefficient; (B) Number of initial infectors; (C) Number of contacted people in office; (D) Number of people in each office; (E) Number of people in each company; (F) Probability of contact between people in different offices in a company; (G) Community size; (H) Number of contacted students in each classroom; (I) Number of students per classroom; (J) Probability of contact between students in different classrooms in a school.
